# Evaluation of Adverse Drug Reactions in Paediatric Patients: A Retrospective Study in Turkish Hospital

**DOI:** 10.3389/fphar.2021.786182

**Published:** 2021-11-12

**Authors:** Zakir Khan, Yusuf Karataş, Olcay Kıroğlu

**Affiliations:** ^1^ Department of Pharmacology, Institute of Health Sciences, Faculty of Medicine, Cukurova University, Adana, Turkey; ^2^ Pharmacovigilance Specialist, Balcali Hospital, Faculty of Medicines, Cukurova University, Adana, Turkey

**Keywords:** adverse drug reactions, children, paediatric, antibiotics, patient safety, Turkey

## Abstract

Drug safety in paediatric patients is a serious public health concern around the world. The paediatric patients are more prone to adverse drug reactions (ADRs) than adults. Moreover, there is a scarcity of information about ADRs in paediatric patients. This study was conducted to determine the frequency, causality, severity, preventability of paediatric patients’ ADRs reported in a tertiary care hospital in Adana, Turkey. A retrospective study was conducted on all spontaneously reported ADRs between January 01, 2020, to July 30, 2021, in paediatric patients. The ADRs reports were evaluated in terms of gender, age, ADR characteristics, suspected drugs and reporting source. All included ADRs reports were characterized according to the Naranjo Algorithm/World Health Organization (WHO) causality scales, Hartwig/Siegel and Common Terminology Criteria for Adverse Events (CTCAE) severity scales, the modified Schoumock and Thornton preventability scale and hospital pharmacovigilance center criteria for seriousness. Therapeutic groups were also coded using the WHO-Anatomical Therapeutic and Chemical (ATC) classification. During the study period, 8,912 paediatric patients who were admitted had 16 ADRs with 1.7 ADRs/1,000 admissions. The majority of ADRs were found in infants (31.2%) and children (56.2%) as compared to adolescents (12.5%). ADRs were observed more in females (81.2%) than males. Skin (62.5%) was the most affected organ due to the ADRs, and maculopapular rash and erythema multiforme were the most commonly reported symptoms. Most ADRs were probable/likely (93.7%), severe (50%), preventable or probably preventable (43.7%) and serious (37.5%). Antibiotics (93.7%) were found to be the most common cause of ADRs in paediatric patients. The majority of ADRs were associated with vancomycin (68.7%). Most of the ADRs were reported by a medical doctor in this study. This small sample size study highlights significant problems of ADRs in paediatric patients, mainly caused by antibiotics and with a majority of ADRs manifest as skin reactions. Furthermore, a high proportion of the identified ADRs were found to be preventable. More focused efforts are needed at the national level to avoid preventable ADRs in hospitals. Monitoring and management of ADRs and future studies would be beneficial for better patient care and safety.

## Introduction

Adverse drug reactions (ADRs) are a leading cause of illness and mortality worldwide (Giardina et al., 2018). Spontaneous reporting of ADRs is critical for effective post-marketing drug surveillance and patient safety (Noda et al., 2020). ADRs are also related to more serious cases of liver injury due to drugs as presented in different databases (Noda et al., 2020; Teschke and Danan, 2021), but many of these liver injury cases were poorly documented and used a global introspection method for causality (Teschke and Danan, 2021). The limitations of these approaches include case selection based solely on published case numbers rather than a strong causality assessment method. Changes in the method of causality assessment of ADRs are required to improve data quality and database reliability. Therefore, an objective approach like Roussel Uclaf Causality Assessment method (RUCAM) should be used to assess causality of ADRs cases for the better outcomes in future studies (Danan and Teschke, 2016; Danan and Teschke, 2019; Teschke and Danan, 2021. Drug safety is an important part of health care and understanding ADRs is crucial for avoiding harmful effects (Dittrich et al., 2020). The safety of drugs in paediatric patients is a serious public health problem (Rosli et al., 2017; Khan et al., 2020a). ADRs in paediatric patients have been shown to cause not only hospital admissions or lengthy hospitalization, but also chronic disability or even death (Le et al., 2006).

The medication mistakes in paediatric patients were found to be three times higher than in adults, mostly due to considerable variation in body mass, which necessitates individual dose measurements depending on patient age, weight, or body surface as well as the clinical situation (Khan et al., 2020a). The development of renal functions and enzyme systems, pharmacokinetic and pharmacodynamic parameters in paediatric patients also alter throughout time (Rosli et al., 2017). Paediatric patients are provided a wide range of medications, with an elevated risk of ADRs linked with off-label prescribing (Bellis et al., 2014). ADRs are responsible for an increased morbidity level in paediatric patients (Priyadharsini et al., 2011; Angamo et al., 2016; Venkatasubbaiah et al., 2018).

Paediatric patients are one of the most vulnerable populations to ADRs. It is reported that ADRs account for nearly 5% of all hospital admissions in paediatric patients (Angamo et al., 2016). A previous study revealed that ADRs affects around one out of every ten children in the hospital, with 12% of them were serious (Clavenna and Bonati, 2009). According to systematic reviews, the overall average incidence of ADRs in paediatric patients was 9.52–9.53% (Impicciatore et al., 2001; Khan et al., 2020a). ADRs also imposed a higher financial cost on patients. It is reported that the average cost of treating an ADR per patient was estimated to be United States dollars (USD) 9,491, with hospitalization or room expenditures accounting for 50% of the total cost (Ayani et al., 1999; Oshikoya et al., 2011). Another study calculated a total cost to a hospital of USD 27,358 for the hospitalization of patients with ADRs in an emergency room over 6 weeks (Patel et al., 2007).

Children rarely articulate their personal medication therapy experiences; they are more susceptible to ADRs. As a result, inappropriate drugs usage in paediatric patients has a significant chance of causing a variety of ADRs (Khan et al., 2020a; Nasso et al., 2020). The paediatric patients are considered susceptible populations and are frequently underrepresented in randomized controlled trials (RCTs). Therefore, there is scarce data about detecting ADRs which provides limited safety information in paediatric patients (Nor-Aripin et al., 2012). To compensate for the limitations of RCTs, spontaneous ADR reporting is an important source of medication safety data in paediatric patients that aren’t often studied in RCTs (Rosli et al., 2017; Gentili et al., 2018).

A statewide, volunteer pharmacovigilance (PV) system exists in Turkey, as in many other nations. Its primary goal is to alert the public about previously unknown risks associated with the use of medications in everyday life (Turkey pharmacovigilance center TÜFAM, 2005). Additional research is usually required to confirm these safety signals. All Health care professionals (HCPs; doctors, nurses, pharmacists, etc.), patients and caregivers are the primary source of voluntary ADRs reporting (Ergün et al., 2019; Khan et al., 2020b; Haines et al., 2020). Under-reporting of ADRs continues to be a widespread issue (Ergün et al., 2019; Güner and Ekmekci, 2019; Khan et al., 2020a). According to a recent report, Turkey submitted only 89 ADR reports per million population to the World Health Organization (WHO) Vigiflow database in 2020 (Turkey Pharmaceuticals and Medical Devices Agency TPMDA, 2020). However, the WHO recommended that ADR reports should be produced at a rate of 200 per million population per year (World health organization WHO, 2021a).

Underreporting is a well-known issue in voluntary ADRs reporting schemes (Hazell and Shakir, 2006; Khan et al., 2020b). Underreporting of ADRs in paediatric patients is also due to a lack of knowledge about adverse reactions to prescribed drugs (Rosli et al., 2017; Khan et al., 2020b; Dittrich et al., 2020). Timely reporting of ADRs and periodic monitoring are useful for improved care and safety in paediatric patients (Dittrich et al., 2020). Moreover, there is a scarcity of information about ADRs in paediatric patients in our healthcare setting and as well in Turkey. Therefore, this study aimed to determine the frequency, causality, severity, preventability of paediatric patients’ ADRs recorded in a tertiary care hospital in Adana, Turkey.

## Materials and Methods

A retrospective study was conducted in a pharmacovigilance center of Balcalı Hospital in Adana, Turkey, to evaluate paediatric patients’ (0–17 years old) ADR reporting forms. Balcalı Hospital is a tertiary care teaching hospital with 1,171 beds that provides both in-patient and out-patient care. It offers health care facilities to the rural and urban population of Adana, (Turkey’s fifth-largest city). This research was carried out per the Helsinki Declaration’s principles. Due to the retrospective nature of the study, involvement of official member of pharmacovigilance center and examination of ADRs reporting forms, the hospital’s institutional ethics committee waived ethical approval. All paediatric patients’ ADRs reported to pharmacovigilance officer by HCPs from January 1, 2020, to July 30, 2021, were included. ADR forms with missing information with unclear causality were omitted from the analysis.

A clinical pharmacologist working at the pharmacovigilance center and researchers analyzed all individual recognized ADR reports. The ADRs reports were evaluated in terms of gender, age, ADR characteristics, suspected drugs, reporting source and outcomes were extracted. The Naranjo algorithm (the total scores range from −4 to 13, the reaction is considered Definite if score >8, probable 5–8, possible 1–4 and doubtful = 0) and WHO-Uppsala Monitoring Centre (the suspected ADR is assessed as certain, probable, possible, unlikely and unclassified/unclassifiable) criteria were used to determine the causality of each suspected ADR (Naranjo et al., 1981; World health organization and Uppsala Monitoring Centre WH/UMC, 2013). These validated tools have been used in several studies (Dittrich et al., 2020; Nasso et al., 2020; Trubiano et al., 2016). Hartwig’s Severity Assessment Scale was used to evaluate the ADRs’ severity. ADRs were classified as mild, moderate, or severe (Hartwig et al., 1992). The severity of the ADRs was also determined by using the Common Terminology Criteria for Adverse Events (CTCAE) scale. For each ADR, the CTCAE displays grades 1 through 5 along with a specific clinical description of severity (Common Terminology Criteria for Adverse Events CTCAE, 2017; Dittrich et al., 2020). The modified Schoumock and Thornton scale was used to assess preventability (ADRs are divided into three categories: definitely preventable, probably preventable and not preventable) (Schumock and Thornton, 1992; Al-Damen and Basheti, 2019). Hospital pharmacovigilance center criteria were used to determine the seriousness of ADR ***.*** Our hospital pharmacovigilance criteria is designed according to the Turkish pharmacovigilance center (TUFAM) recommendations. The hospital pharmacovigilance officer assessed the nature and outcome of ADR after follow-up and classify the cases as “death, life-threatening, caused hospitalization/prolonged hospitalization, caused permanent disability, Other (any other adverse effect due to a drug) and then forward the form to TUFAM and also discussed with HCPs. This form shows the outcome of ADRs experienced by patients. Therapeutic groups and drugs were also coded using the WHO-Anatomical Therapeutic and Chemical (ATC) classification (World Health organization (WHO), 2020b).

SPSS Statistics version 25 (IBM Corp., SPSS Statistics) was used to perform a descriptive analysis of the data for frequency, mean, and percentage. All of the information is presented in tabular and graphical formats.

## Results

A total of 29 ADRs were submitted to the hospital pharmacovigilance center during 2020–2021. Of these 17 (58.6%) were related to paediatric patients. One ADR form was excluded due to the missing information. Finally, 16 eligible ADRs forms (11 reports during 2020 and 5 in 2021) were analyzed in this study. According to the hospital data, a total of 8,912 (4,701 in 2020 and 4,211 in 2021) paediatric patients were admitted during the study period with 1.7 ADRs/1,000 admissions. These ADRs were observed during hospital admission and reported to the hospital pharmacovigilance center. The majority of ADRs were found in infants (31.2%) and children (56.2%) as compared to adolescents (12.5%). ADRs were observed more in females (81.2%) than males. The median age of the patients was 6.5 years (2 months–13.5 years).

According to the Naranjo algorithm/WHO-UMC scales, 15 of the ADRs were probable/likely (93.75%) and 1 was definite/certain (15.8%). Hartwig’s Severity Assessment Scale shows that 3 of the ADRs were mild (18.75%), 5 were moderate (31.25%) and 8 were severe (50%) requiring an intensive medical intervention. The CTCAE criteria showed that five ADRs were grade 2, four ADRs were grade 3, and seven ADRs were grade 4. According to the modified Schumock and Thornton scale, 7 ADRs were measured as preventable (43.5%) and among them, four were “definitely preventable” (25%) and three were “probably preventable” (18.75%). The remaining (*n* = 9; 56.25%) were “non-preventable”. Moreover, according to the hospital pharmacovigilance center criteria, 9 (56.25%) of the ADRs caused hospitalization/prolonged hospitalization and 6 (37.5%) were life-threatening (Table 1).

**TABLE 1:** Characteristics and assessment of paediatric patients ADR reports.

	—
Gender	n (%)
Male	3 (18.75)
Female	13 (81.25)
Age groups	
Neonates (birth to 1 month)	0 (0)
Infants (>1 month to 2 years)	5 (31.25)
Children (>2–12 years)	9 (56.25)
Adolescents (>12–17 years)	2 (12.5)
Year of reporting	
2020	11 (68.75)
2021	5 (31.25)
Causality	
Naranjo algorithm	—
Probable	15 (93.75)
Definite	1 (6.25)
WHO/UMC	—
Likely	15 (93.75)
Certain	1 (6.25)
Severity	
Modified Hartwig/Siegel scale	—
Level 2 (mild)	3 (18.75)
Level 4 (moderate)	5 (31.25)
Level 5 (severe)	8 (50)
CTCAE scale	—
Grade 2 (moderate)	5 (31.25)
Grade 3 (severe)	4 (25)
Grade 4 (life-threatening)	7 (43.75)
Preventability	
Definitely preventable/Probably preventable	7 (43.75)
Not preventable	9 (56.25)
Seriousness criteria (Hospital criteria)	
Death	0 (0)
Life-threatening	6 (37.5)
Caused hospitalization/prolonged hospitalization	9 (56.25)
Caused permanent disability	0 (0)
Other	1 (6.25)
System organ class	
Skin	10 (62.5)
Renal and urinary disorders	4 (25)
Circulatory system	2 (12.5)
Management	
The medication was stopped	9 (56.25)
Another medication was substituted	1 (6.25)
Dose was reduced	2 (12.5)
Another medication was added to combat adverse effect	4 (25)
Outcomes	
Recovered	14 (87.5)
Recovering	2 (12.5)
Fatal	0 (0)

Abbreviation ADRs = Adverse drug reactions, n = frequency, % = percentage, WHO/UMC = World health organization-Uppsala monitoring centre, CTCAE = Common terminology criteria for adverse events.

Skin (62.5%) and renal system (25%) were the most affected organs due to the ADRs. The suspected medication was discontinued in 10 (62.5%) of the patients and replaced with another medication for the same indication. The dose of suspected medication was reduced in 2 (12.5%) patients to alleviate symptoms, while another drug was given to overcome adverse effects in 4 (25%) cases. The results of ADR management revealed that 87.5% had been recovered and 12.5% were in the process of recovering. No fatal case due to ADR was reported in the current study (Table 1).

In our study, ADRs were mostly associated with antibiotics (15; 93.75%) followed by antiviral (*n* = 1, 6.25%). Vancomycin (*n* = 11, 68.75%) was associated with the highest number of ADRs. The most common reported ADRs with vancomycin use were maculopapular rash (*n* = 5, 31.25%) and anaphylaxis (*n* = 2, 12.5%). Antiviral drug (Aciclovir) is associated with acute kidney failure. The most common drugs that cause ADRs, as well as associated reactions, are listed in Table 2.

**TABLE 2:** Drugs associated with ADRs.

Drug class	WHO/ATC code	n (%)	Reaction details (no of patients)
Antibiotic			
Vancomycin	J01XA01	11 (68.75)	Maculopapular rash (5)
—	—	—	Anaphylaxis (2)
—	—	—	Erythema multiforme/Redness on the whole body (2)
—	—	—	Acute kidney injury (1)
—	—	—	Increase creatinine level (1)
Clindamycin	J01FF01	1 (6.25)	Maculopapular rash (1)
Colistin-Linezolid	J01XB01-J01XX08	1 (6.25)	Increase creatinine level (1)
Ceftriaxone	J01DD04	1 (6.25)	Rashes and urticaria (1)
Amphotericin B	J02AA01	1 (6.25)	Maculopapular rash (1)
Antiviral			
Aciclovir	J05AB01	1 (6.25)	Acute kidney injury (1)

Abbreviation ADRs = Adverse drug reactions, n = frequency, % = percentage, WHO/ATC = World health organization/Anatomical therapeutic and chemical classification.

These ADRs were first time reported in the hospital pharmacovigilance center. All of the ADRs were reported by doctors (*n* = 15, 93.75%) and nurses (*n* = 1; 6.25%). We did not observe any ADR reported by a pharmacist and other paramedical staff in this study.

## Discussion

Periodic evaluation of ADRs in paediatric patients is very important. This study explored the causality, preventability, severity of ADRs as well as therapeutic groups and reactions related to ADRs. The current retrospective study is the first attempt that has been conducted to analyze the paediatric patients’ ADRs in our healthcare setting. As a result, it could serve as a baseline for future study, as well as provide critical evidence for healthcare stakeholders and government decision-makers to take the necessary steps to reduce the burden of ADRs in paediatric patients.

In our study, out of the total reported ADRs in hospital pharmacovigilance centers, 58.6% were related to paediatric patients. Similar studies conducted in Netherlands (Dittrich et al., 2020) and Malaysia (Rosli et al., 2017) observed that 26 and 14% of admitted paediatric patients had ADRs, respectively. Moreover, the observed rate of ADR related to paediatric patients was 1.7 ADRs per 1,000 admissions. Nasso et al. also reported similar findings (1.6 ADRs/1,000 admissions) in paediatric patients (Nasso et al., 2020). However, another study conducted by Lombardi et al., observed 2.2 ADRs per 1,000 paediatric admissions, which was higher as compared to our study (Lombardi et al., 2018). These differences in ADR rates among studies may be due to variation in data collection methods, sample size, methodology, and healthcare setting.

Causality analysis is necessary to understand the factors that contribute to the occurrence of ADRs (Venkatasubbaiah et al., 2018). In the current study, the Naranjo algorithm and WHO-UMC scales of causality assessment observed that most of the ADRs were probable/likely categories. Similar findings were also reported by Nasso et al., 2020, Al-Damen and Basheti, 2019, and Saqib et al., 2018). Because of their simplicity, the Naranjo algorithm and WHO-UMC scales are also used to evaluate the causality score in paediatric patients. We used Naranjo and the WHO method because these are suitable for non-hepatic ADRs, but not for hepatic ADRs (García-Cortés et al., 2011; Teschke et al., 2013; Danan and Teschke, 2016; Lin et al., 2019). Therefore, in future studies showing paediatric patients with suspected drug-induced liver injury (DILI) or herb-induced liver injury (HILI), all cases should be assessed for causality using the updated RUCAM published in 2016 (Danan and Teschke, 2016; Danan and Teschke, 2019).

In the current study, according to the severity assessment of ADRs using the Modified Hartwig and Siegel scale, the majority of ADRs fell into the severe followed by moderate and mild categories. However, these findings deviated from the studies conducted in Malaysia (Rosli et al., 2017) and India (Sundaran et al., 2018) which disclosed a higher ADRs proportion as mild and moderate while a small number were severe. In addition, CTCAE criteria showed that the majority of ADRs were grade 3 (severe) and 4 (life-threatening) categories in this study. Similar findings were also reported in the Netherlands study (Dittrich et al., 2020). The severity of ADRs must be assessed to take serious steps against the drug’s continued use. Severe ADRs have been linked to a longer stay in the hospital and a higher financial cost due to the need for more intensive medical care. (Walter et al., 2017; Fasipe et al., 2019).

In this study, preventability assessment using Modified Schumock and Thornton scale showed 43.7% of ADRs were definitely and probably preventable. Supported findings were also reported in the Jordan study and reported that 44.7% of ADRs were definitely and probably preventable (Al-Damen and Basheti, 2019). Another study conducted among paediatric patients observed that 20% of ADRs were preventable or probably preventable (Nasso et al., 2020). In the current study, most of the identified ADRs were found to be preventable. The establishment of active ADR surveillance and raise awareness is important to encourage safer drug use. Periodic ADR reporting programs are required to educate and enhance awareness among all HCPs. More focused efforts are needed at the national level to avoid preventable ADRs in hospitals. Therefore, ADRs should be monitored carefully to avert hazardous effects.

In the present study, antibiotics were the most common drug class followed by antiviral reported for ADRs in paediatric patients. Similar results were detected by previously published studies (Rashed et al., 2012; Rosli et al., 2017). The most frequently involved antibiotics associated with ADRs were Vancomycin followed by Ceftriaxone, Clindamycin and Colistin-Linezolid in our study. A study conducted among paediatric patients reported Amoxicillin and beta-lactamase inhibitors as frequently contributed to ADRs (Nasso et al., 2020). A large number of antibiotics were prescribed in the general paediatric patients, and a higher number of ADRs were reported for drugs in this treatment group (Gallo et al., 2012; Nasso et al., 2020; Priyadharsini et al., 2011). Due to the problem of antibiotic resistance, paediatric patients infections were frequently treated with a combination and broad-spectrum of antibiotics at high doses (Manan et al., 2016).

The skin was the most affected organ by ADRs in this study. Similar findings were also reported by previously published studies (Priyadharsini et al., 2011; Rosli et al., 2017; Nasso et al., 2020). However, a study conducted by Dittrich and colleagues observed that gastrointestinal disorders were more frequently seen in paediatric patients (Dittrich et al., 2020). The reason for increased cutaneous involvement of ADRs in paedriatric patients can be attributed to this age group’s unique physiology; in fact, they have a partially matured epidermis that is not fully developed (Rashed et al., 2012; Rosli et al., 2017). As a result, the skin becomes more porous and vulnerable to chemical and microbial attacks (Stamatas et al., 2011).

In this study, the majority of the antibiotics were linked to ADRs related to skin reactions, which is consistent with the findings of other studies (Priyadharsini et al., 2011; Rosli et al., 2017). Maculopapular rashes followed by anaphylaxis and redness on the whole body (erythema multiforme), acute kidney injury were the main ADRs of Vancomycin observed in our study. Al-Damen and Basheti were also reported acute kidney injury with Vancomycin use (Al-Damen and Basheti, 2019). In addition, erythema multiforme has been linked to a variety of antibiotics, including b-lactams, macrolides, aminoglycosides, glycopeptides, and others (Diaz and Ciurea, 2012). According to the previously published study, the main Vancomycin-related ADRs were skin rashes and elevated serum creatinine (An et al., 2011). Another study also reported anaphylaxis to intravenous Vancomycin in paediatric patients (Xie et al., 2021). Close monitoring of laboratory testing, including complete blood counts with differential analysis, is recommended for the early and precise diagnosis of ADRs associated with Vancomycin use (An et al., 2011; Xie et al., 2021).

Antifungal antibiotic drug (Amphotericin B) caused rashes in one paediatric patient in this study. A study conducted in India observed renal failure and diarrhea as ADRs due to Amphotericin-B use (Sundaran et al., 2018). It is reported that prolonged Amphotericin-B treatment can be associated with maculopapular rash, eosinophilia and also systemic symptoms (Cesaro et al., 1999; Hagihara et al., 2015). Careful monitoring of the patient being treated for the first time is warranted in the case of Amphotericin B. Antiviral drug (Aciclovir) was responsible for Acute kidney injury-related ADR in our study. Acyclovir is an antiviral medicine that is commonly prescribed to paediatric patients, and it can cause acute kidney injury (Fleischer and Johnson, 2010). A recent study also reported that an acute renal injury occurred in 13% of parenteral Aciclovir treatment episodes (Ryan et al., 2018). Therefore, dosage adjustments for baseline renal function and optimal body weight are crucial to prevent ADRs (Yildiz et al., 2013).

In the current retrospective study, no DILI case was reported. A previously published study conducted by Zhu et al. observed serious conditions of DILI in children and antibiotics were the most commonly reported drug class with risk of liver injury (Zhu et al., 2015). Limited data is available regarding DILI cases in paediatric patients. It is reported by Shi et al. that DILI in children accounts for about 1% of all reported ADRs throughout all age groups, less than 10% of all clinical DILI cases, and around 20% of all acute liver failure cases in children. (Shi et al., 2017). DILI is one of the most serious ADRs and accounts for 13% of all cases of acute liver failure in the United States (Ostapowicz et al., 2002). Moreover, HILI also accounts for 24.2% of the study cohort consisting of both, DILI and HILI cases in China (Li et al., 2007), 11% in Spain (Andrade et al., 2005) and 0.60% in Korea (Cho et al., 2017). Various studies also reported DILI and HILI cases in adults (Mitchell and Hilmer, 2010; Cho et al., 2017; Teschke and Danan, 2017; Amadi and Orisakwe, 2018; Becker et al., 2019).

Many of the used drugs in our study also may cause DILI such as previously reported with antibiotics and other therapeutic drugs (Danan and Teschke, 2016; Teschke, 2018; Zhang et al., 2019; Teschke and Danan, 2020), confirming previous data of various RUCAM based DILI cases caused by multiple drugs as published in worldwide cases (Teschke and Danan, 2017; Teschke, 2018; Teschke and Danan, 2020). The underline reasons for lack of DILI and HILI in our study may be that the reporting rate of ADRs to the pharmacovigilance center was very low. We observed all reported ADRs during the study period however no ADR case was reported due to DILI and HILI. Low reporting rate of ADRs is the main reason as indicated in previous studies (Ghabril et al., 2010; Cho et al., 2017; Teschke and Danan, 2017). The risk of liver injury due to medication in children should be taken seriously, and particular emphasis should be placed on the risk of liver injury caused by drugs (Zhang et al., 2019). Therapeutic monitoring of drugs and assessment of liver tests indicators are necessary for timely and correct diagnosis of liver injury (Lee and Senior, 2005; Avigan et al., 2014). However, liver tests were mentioned in only 3 cases of vancomycin in the reported ADR form in our study and none of the patients did have increased Aspartate transaminase (AST) and Alanine transaminase (ALT) levels. Therefore, more active surveillance training on ADRs for all healthcare professionals (HCPs) is crucial for proper monitoring of ADRs in children to avoid the risk of liver injure and better patient safety.

Most of the ADRs were reported by a medical doctor in this study. Nurses reported only one ADR and reporting from the pharmacist was not observed. The accurate spontaneous ADRs reporting mechanisms used by prescribers, nurses, pharmacists, and other paramedical workers are critical for the detection of serious ADRs in hospitals (Giardina et al., 2018; Güner and Ekmekci, 2019). ADR underreporting is a global issue that has been documented in previous international studies (AlShammari and Almoslem, 2018; Alwhaibi and AlAloola, 2020). One of the Turkish government’s major priorities is to monitor and report ADRs (Turkey Pharmaceuticals and Medical Devices Agency TPMDA, 2014). In 2005, Turkey established the “Turkish Pharmacovigilance Center (Turkish: Türkiye Farmakovijilans Merkezine)” to coordinate PV activities across the country. In Turkey, all HCPs are expected to be vigilant in identifying and reporting ADRs to the hospital pharmacovigilance center or directly to TUFAM (Ergün et al., 2019; Khan et al., 2020b). However, despite the potential hazards of ADRs and the implementation of WHO standard pharmacovigilance in Turkey, the under-reporting of ADRs continues to be a widespread issue (Ergün et al., 2019; Güner and Ekmekci, 2019; Khan et al., 2020b; Turkey Pharmaceuticals and Medical Devices Agency TPMDA, 2020). Previously published studies in Turkey as well as at a global level reported that HCPs have insufficient knowledge about pharmacovigilance systems and ADRs reporting (Ergün et al., 2019; Güner and Ekmekci, 2019; Alwhaibi and AlAloola, 2020; Nadew et al., 2020). Lack of knowledge about adverse reactions to prescribed drugs in paediatric patients is also responsible for underreporting of ADRs (Rosli et al., 2017; Khan et al., 2020b; Dittrich et al., 2020). Therefore, the creation of a mandatory unified periodic education intervention on ADRs of drugs is crucial for better paediatric patients care. Turkish health policymakers should also emphasize the importance of adequate cooperation between international, local health authorities and manufacturers to stimulate and support regular joint training programs for all HCPs to increase ADR knowledge and reporting.

Our research contains both limitations and strengths. This is a single-center study involving only paediatric patients; therefore, it cannot be generalized to other healthcare settings across the country. Moreover, a small number of identified ADRs (*n* = 16) were evaluated due to a low reporting rate in hospital pharmacovigilance centers. Lack of training, inaccessibility to ADR reporting forms, poor skills, time restrictions, and a lack of incentives are all plausible reasons for HCPs underreporting (Venkatasubbaiah et al., 2018; Khan et al., 2020b). There may be also a chance of unreported mild ADRs due to the voluntarily reported system in the hospital which may pose some underestimation of ADRs. Therefore, a multicenter study and active surveillance of ADRs with a large sample size are required to further verify the current study’s findings. The retrospective nature of the study may have been limited data accuracy and information. However, almost all of the information was obtained from registered ADR reports available in a hard form in the hospital pharmacovigilance center. We did not observe any DILI cases in this study. The possible reason may be a small number of reported ADRs and a lack of information regarding liver tests on ADRs forms. However, it is confirmed that three patients had liver tests reports but none of the patients did have increased liver tests such as AST and ALT. There is a need for continuous periodic training on ADRs surveillance to monitor medication in children to avoid the risk of liver injury. Moreover, our findings on the most commonly reported drug and clinical symptoms may be divergent from other populations with different prescribing patterns, disease epidemiology, and ethnicities. On the other hand, our study highlights the importance of a pharmacovigilance monitoring system to improve quality reporting. Periodic monitoring of ADRs is useful for paediatric patients’ safety and also for additional literature data, which is currently rare. Moreover, this is the first study of ADRs among paediatric patients in our hospital. As a result, it could serve as a starting point for future research and give essential evidence for healthcare stakeholders and government decision-makers to take the required actions to lessen the burden of ADRs in paediatric patients.

## Conclusion

This small-scale retrospective study revealed the current ADRs pattern in paediatric patients. Antibiotics were the leading cause of ADRs, which may reflect the widespread use of antibiotics in this population. The majority of ADRs were related to skin reactions, and a considerable proportion was preventable types. More focused efforts are needed at the national level to avoid preventable ADRs in hospitals. Monitoring and management of ADRs and future studies would be valuable for improved patient care and safety. Moreover, a large sample size and multi-central studies are needed to validate the current study results and highlight the area for further improvement in different healthcare settings.

## Data Availability

The original contributions presented in the study are included in the article/Supplementary Material, further inquiries can be directed to the corresponding author.
