# Corrigendum: Evaluation of Adverse Drug Reactions in Paediatric Patients: A Retrospective Study in Turkish Hospital

**DOI:** 10.3389/fphar.2021.813892

**Published:** 2021-12-14

**Authors:** Zakir Khan, Yusuf Karataş, Olcay Kıroğlu

**Affiliations:** ^1^ Department of Pharmacology, Institute of Health Sciences, Faculty of Medicine, Cukurova University, Adana, Turkey; ^2^ Pharmacovigilance Specialist, Balcali Hospital, Faculty of Medicines, Cukurova University, Adana, Turkey

**Keywords:** adverse drug reactions, children, paediatric, antibiotics, patient safety, Turkey

In the published article, there was an error in the affiliation for author **Olcay Kıroğlu**. Instead of “Pharmacovigilance Specialist, Balcali Hospital, Faculty of Medicines, Cukurova University, Adana, Turkey,” it should be *Department of Pharmacology, Institute of Health Sciences, Faculty of Medicine, Cukurova University, Adana, Turkey.*

The authors apologize for this error and state that this does not change the scientific conclusions of the article in any way. The original article has been updated.

